# A novel submerged *Rotala rotundifolia*, its growth characteristics and remediation potential for eutrophic waters

**DOI:** 10.1038/s41598-019-51508-y

**Published:** 2019-10-16

**Authors:** Chaoguang Gu, Feifei Li, Jibo Xiao, Shuyi Chu, Shuang Song, Ming Hung Wong

**Affiliations:** 1 College of Environment, Zhejiang University of Technology, Hangzhou, 310032 China; 2 Collaborative Innovation Center of Yangtze River Delta Region Green Pharmaceuticals, Zhejiang University of Technology, Hangzhou, 310032 China; 3 College of Life and Environmental Science, Wenzhou University, Wenzhou, 325035 China; 4 Wenzhou Vocational College of Science and Technology, Wenzhou, 325000 China; 5 Guangdong Provincial Key Laboratory of Soil and Groundwater Pollution Control, and State Environmental Protection Key Laboratory of Integrated Surface Water-Groundwater Pollution Control, Southern University of Science and Technology, Shenzhen, Guangdong 5188055 China; 6 Consortium on Health, Environment, Education and Research (CHEER), Department of Science and Environmental Studies, The Education University of Hong Kong, Tai Po, Hong Kong China

**Keywords:** Restoration ecology, Environmental sciences

## Abstract

The vegetative growth and remediation potential of *Rotala rotundifolia*, a novel submerged aquatic plant, for eutrophic waters were investigated on different sediments, and under a range of nitrogen concentrations. *Rotala Rotundifolia* grew better on silt than on sand and gravel in terms of plant height, tiller number and biomass accumulation. Percent increment of biomass was enhanced at low water nitrogen (ammonium nitrogen concentration ≤10 mg/L). The maximum total nitrogen and total phosphorus removals in the overlying water were between 54% to 66% and 42% to 57%, respectively. Nitrogen contents in the sediments increased with increasing water nitrogen levels, whereas, nitrogen contents in the plant tissues showed no apparent regularity, and the greatest value was obtained at ammonium nitrogen concentration 15 mg/L. Both phosphorus contents in the sediments and tissues of plants were not affected significantly by additional nitrogen supply. Direct nitrogen uptake by plants was in the range of 16% to 39% when total phosphorus concentration was 1.0 mg/L. These results suggested that *Rotala Rotundifolia* can be used to effectively remove nitrogen and phosphorus in eutrophic waters.

## Introduction

Eutrophication resulted from excessive discharge of nutrients is still the most prevalent water problem in many countries all over the world^1^. Major nutrient sources include agricultural runoff^2^, domestic sewage^3^, industrial effluents^4^ and atmospheric dry and wet deposition^5–7^. High nutrient loads in water may cause rapid growth of algae, consequently, reduce or eliminate dissolved oxygen (DO) in the water, leading to death of large numbers of fish^8,9^. Most waters lost its primary functions due to deterioration of water quality and changes in ecological structures. Therefore, reduce and control nutrients in water is still an urgent issue^10,11^.

Submerged macrophytes are important components of freshwater ecosystem. They inhibit abundant growth of algae^12^, increase water transparency^13^, and provide food resources and habitats for aquatic animals^14^. The stems, leaves and epidermis of submerged macrophytes are capable of assimilating nutrients as roots^15^. Besides, they can retain sediments and limit the resuspension of nutrients to overlying water^16^. Thus, the nutrient levels are generally lower in waters dominated by submerged macrophytes^17^. The re-establishment of submerged vegetation is recognized as an efficient approach for improving water quality and restoring ecological functions of degraded aquatic ecosystem^18^.

However, many submerged macrophytes grow seasonally, and would die away. For example, *Myriophyllum spicatum*, *Ceratophyllum demersum*, and *Vallisneria natans* could not live through the winter, while *Elodea nuttallii* and *Potamogeton crispus* slow down their growth in June and gradually die away^19^. The decay process of the biomass releases nutrients that have been incorporated into the plant tissues to the overlying water again^20^. Furthermore, the debris of the plants would settle to the bottom of the water, leading to the pollution of sediments. Therefore, exploring and cultivating new species is of the great significance for ecological restoration and submerged aquatic plant diversity.

*Rotala rotundifolia* is a perennial aquatic plant with soft stems that branch often to form low, creeping clumps. This species has both submerged and emergent forms, which differs in a number of characteristics. The emergent form has fleshy, bright-green and rounded leaves, while the submerged has darker green or reddish leaves that are thin and lanceolate sword-shaped. It is not considered cold-tolerant, suitable for tropical aquariums with water temperature below 30 °C, but not lower than 21 °C^21^. However, the submerged *Rotala rotundifolia* found in the remote valley in Wenzhou city, Zhejiang Province, China, is able to survive across the winter under the temperature as low as 4 °C. Few reports were available on the growth characteristics and nutrient removal potential of this species. Therefore, the present study focused on the growth responses of this novel *Rotala rotundifolia* on different sediments (gravel, sand and silt), various concentrations of N (3, 5, 7, 10, and 15 mg/L), and the remediation performance for eutrophic waters with the aim of better understanding the characteristics of *Rotala rotundifolia* and providing data for its further application.

## Materials and Methods

### Growth characteristics on different sediments

**Figure 1:**
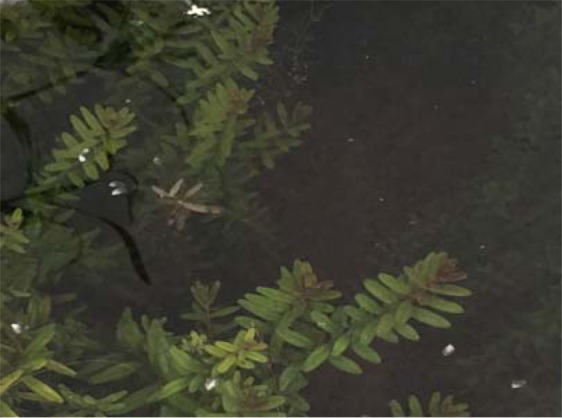
Submerged *Rotala Rotundifolia*.

**Figure 2:**
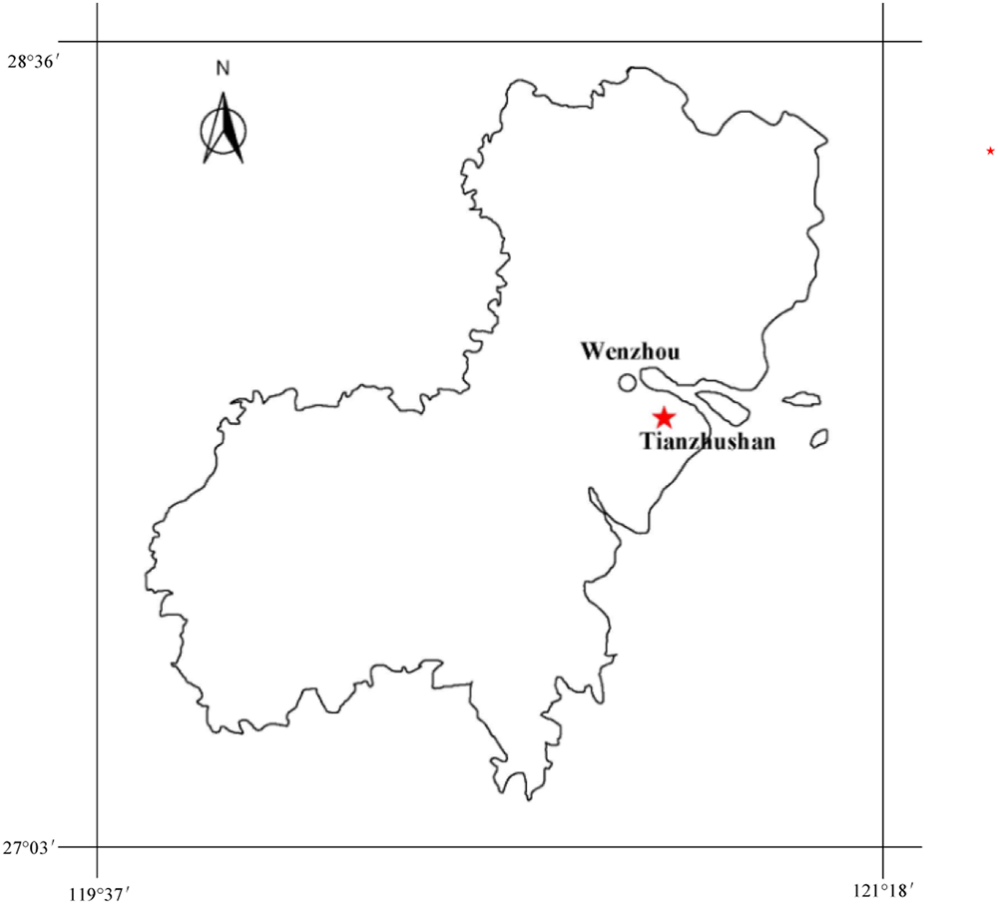
Map of Tianzhushan area in Wenzhou city, China (drawn by autocad 2007 software).

*Rotala rotundifolia* (Fig. 1) was collected from the remote valley in Tianzhushan in Wenzhou City, China (Fig. 2), and rinsed to remove invertebrate grazers and cultured in the experimental plot for 45 days. Then the sturdy plants (approximately 45 cm in length) were transplanted to the batch cultures in plastic tanks (upper diameter 30.5 cm, lower diameter 25.5 cm, height 32 cm), containing 15 L of the eutrophic water. A stock solution of NH_4_Cl and KH_2_PO_4_ and 2% N and P free Hoagland solution were used to prepare the eutrophic water. Initial concentrations of ammonium nitrogen (NH_4_^+^-N) and total phosphorus (TP) were 5.0 mg/L and 0.3 mg/L, respectively. The composition of the Hoagland solution was based on Epstein^22^ as follows (in mM): K (KCl) 6, Ca (CaCl_2_) 4, Mg (MgSO_4_·7H_2_O) 1, B (H_3_BO_3_) 0.025, Mn (MnSO_4_·H_2_O) 0.002, Zn (ZnSO_4_·7H_2_O) 0.002, Cu (CuSO_4_·5H_2_O) 0.0005, Mo (H_2_MoO_4_) 0.0005, Fe (EDTA·FeNa) 0.02. Gravel, sand, and silt were selected in the experiment as they were typical sediments in waters: treatment 1 was gravel (5–8 mm); treatment 2 was sand (0.4–0.9 mm); treatment 3 was silt, each with triplicates. The silt was the dredged silt obtained from the river in November 2015. After being air-dried, silt was sieved through a 0.15 mm sieve to remove coarse debris, and mixed thoroughly. Nitrogen and phosphorus contents of the silt were 2.2 and 0.82 g/kg, respectively. The trial was conducted under natural light from 25 November 2015 to 16 January 2016 in a double-poly greenhouse. During the experiment, losses in water volume due to evaporation of treatment and control plots were compensated by addition of deionized water to the original level every other day. The length and tiller number of the plants, DO of the water were recorded every 10 days. The fresh weight of the plants was analyzed before and at the end of the trial.

### Growth and purification performance on different water N levels

The sturdy plants (approximately 45 cm in length) were transplanted to the batch cultures in plastic tanks (upper diameter 30.5 cm, lower diameter 25.5 cm, height 32 cm), containing 15 L of the eutrophic water with various concentrations of NH_4_^+^-N. Five N levels (3, 5, 7, 10, and 15 mg/L) were used. Thus, there were five treatments, each with triplicates, while no plant was maintained in the control plots. Silt was adopted as the sediment in the trial. Water samples were collected every 3 days from the control and the treatment plots and analyzed for water quality parameters, including pH, temperature, concentrations of NH_4_^+^-N, nitrate nitrogen (NO_3_^−-^N), total nitrogen (TN), TP, and DO. Fresh mass of plants, N and P concentrations in the tissues and sediments were analyzed before and after the trial. Percentages removal of N and P shown in the figures were calculated by the values of treatment groups subtract those of control.

### Analytical methods

Total nitrogen of the water sample was determined by a simultaneous total organic carbon/TN analyser (multi N/C 2100, Analytik Jena, Germany). Nitrate nitrogen was detected by ion chromatography (ICS1500, USA). Ammonium nitrogen was measured according to the Chinese SEPA Standard Methods^23^. Dissolved oxygen and pH were estimated using a portable DO meter (HI9147-04, Hanna, Italy) and a pH meter (FG2-ELK, Mettler Toledo, USA), respectively. The length of the plant was measured by a ruler. Tiller number was counted. Plants were washed with tap and distilled water, and subsequently oven dried at 65 °C for 48 h. The sediments were oven dried at 85 °C for 24 h. Dried plants and sediments were ground into powders. The subsamples of plants and sediments were digested according to the sulfuric acid-hydrogen peroxide (H_2_SO_4_-H_2_O_2_) and perchloric-sulfuric acid (HClO_4_-H_2_SO_4_) method^24^ respectively, and analyzed for TP concentration using colorimetry (λ = 700 nm). As for nitrogen, both subsamples were digested using sulfuric-accelerator, and detected by fully automatic kjeldahl nitrogen analyzer (K370, Buchi, Swiss). The percentage removal efficiency was determined with the following equation: (initial concentration of NH_4_^+^-N, TN, or TP−concentration of NH_4_^+^-N, TN, or TP after t days) (mg/L)/(initial concentration of NH_4_^+^-N, TN, or TP) (mg/L) × 100. The percentage uptake of N or P by plant was calculated as: net uptake content of N or P by plant (mg)/initial TN or TP content in the eutrophic water (mg) × 100.

### Statistical analysis

The results were presented as their mean and standard deviation (SD). All data analyses were conducted using SPSS software (ver.13.0). One-way ANOVA was used to determine significant differences at a significance level of p < 0.05. Post hoc comparisons were done using Turkey HSD test.

## Results

### Growth and morphology on different sediments

**Figure 3:**
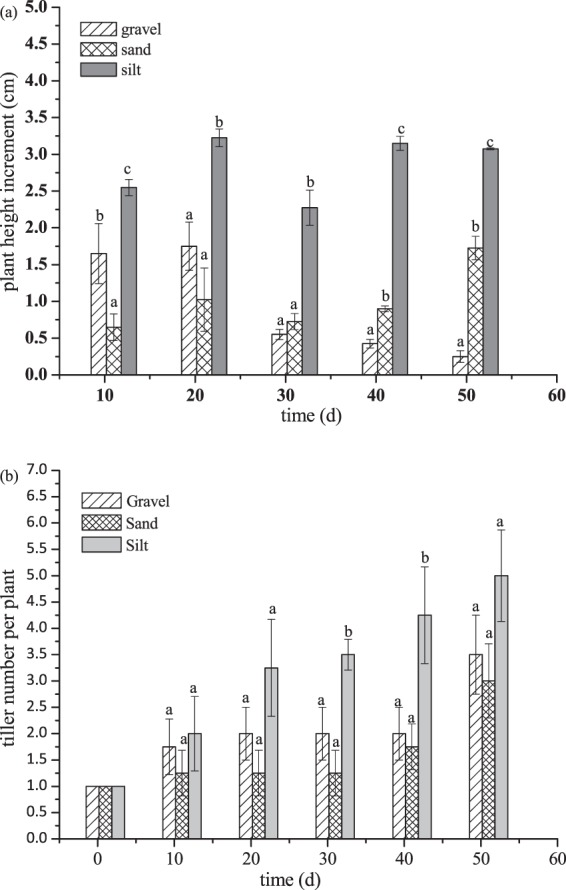
Plant height increment and tiller number per plant of *Rotala Rotundifolia* on silt, gravel and sand. Bars sharing the same letters are not significantly different at P = 0.05. Values are mean ± SD (n = 3).

Plant height was quite different in five measuring time intervals among three types of sediments (Fig. 3a). The height increment on silt was significantly higher than on gravel and sand. At the end of experiment, average height on silt was 60.58 cm, with an increase of 31%, significantly greater than those on gravel (11%) and sand (10%). There was no apparent trend in the plant height increment on silt, which was slightly higher in the second, fourth and final measuring time intervals. The average height increase rate was 0.28 cm·d^−1^. On gravel, the plant height increased fast during the first two measuring time intervals, whereas quite slow in the latter period of the experiment. The plant height increment showed no significant difference (p > 0.05) among the first four measuring time intervals on sand, and it was greatest at the last measuring time. Their corresponding height increase rates were 0.09 and 0.10 cm·d^−1^, respectively.

**Figure 4:**
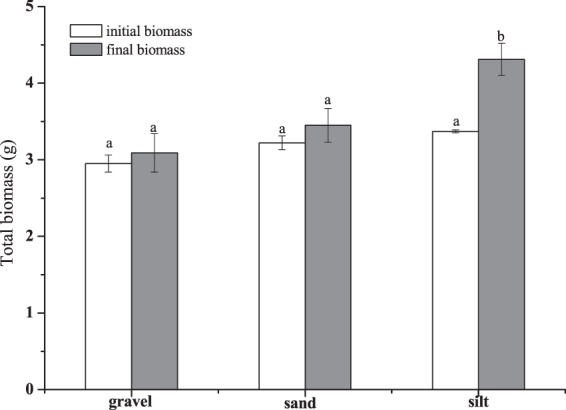
Initial and final biomass of the plant on silt, gravel and sand. Bars sharing the same letters are not significantly different at P = 0.05. Values are mean ± SD (n = 3).

Tiller emergence was observed on all three types of sediments (Fig. 3b). On silt, the tiller number increased gradually along with time, and was significantly higher than on gravel and sand at day 30 and 40. Tiller production was slow during the initial 40 days on both gravel and sand, however increased markedly in the last 10 days of the experiment. For average, the tiller number per plant was 5.0, 3.5, 3.0 on silt, gravel and sand, respectively at the end of experiment, and the difference was not significant. Biomass accumulation was positively relative to plant height. It was greatest on silt, with an increase of 28%, followed by on sand of 7.1%, and on the gravel of 4.8% (Fig. 4). The biomass of plant grown on silt was significantly higher than on gravel and sand at the end of experiment.

### Nutrient removal on silts with different water N supply

#### Plant growth

Total biomass before and after the experiment was analyzed, and the biomass accumulation ratio was also calculated, the results are shown in Table 1. Total biomass increased gradually with water N supply in the range of 3 to 10 mg/L, but decreased rapidly with further increase of NH_4_^+^-N concentration. When NH_4_^+^-N concentration was 15 mg/L, the total biomass was 114.2 ± 12.6 g, with the accumulation ratio of 14%, which was slightly lower than the value at NH_4_^+^-N concentration of 3 mg/L.

**Table 1:** Total biomass of *Rotala Rotundifolia* before and after the experiment at different NH_4_^+^-N concentrations. Values are mean ± SD (n = 3).

NH_4_^+^-N concentration (mg/L)	Initial biomass (g)	Final biomass (g)	Percent increment (%)	Uptake by plants (%)	Synergistic effect of plants (%)	Contribution of other effects (%)
3	99.2 ± 0.5	113.7 ± 6.1	15	26 ± 7.7	13 ± 8.0	58 ± 0.19
5	99.4 ± 0.4	115.0 ± 2.1	16	28 ± 5.8	11 ± 5.7	59 ± 1.3
7	98.3 ± 0.8	128.1 ± 18.8	30	39 ± 4.8	9.4 ± 1.4	49 ± 2.1
10	100.0 ± 0.9	132.5 ± 8.2	32	31 ± 8.1	28 ± 8.1	39 ± 1.0
15	100.0 ± 1.0	114.2 ± 12.6	12	16 ± 10	39 ± 7.2	34 ± 0.59

### Nitrogen removal in the overlying water

**Figure 5:**
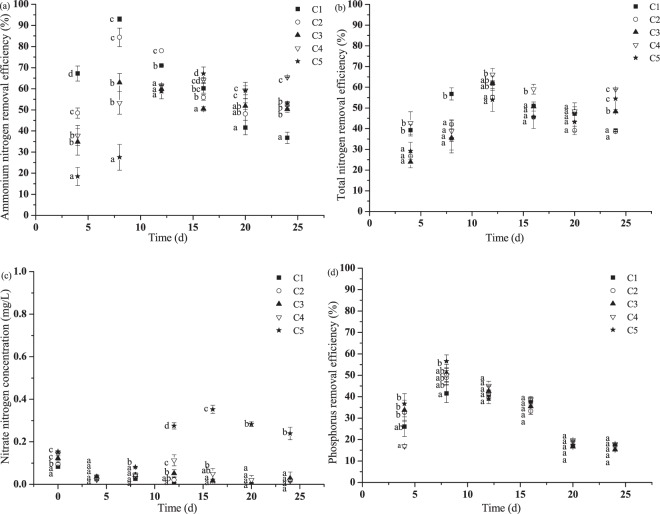
(**a**–**d**) Nitrogen and P removals in the overlying water at different NH_4_Cl concentrations (C1: 3 mg/L; C2: 5 mg/L; C3: 7 mg/L; C4: 10 mg/L; C5: 15 mg/L). (**a**) Ammonium nitrogen removal efficiency. (**b**) Total nitrogen removal efficiency. (**c**) Nitrate nitrogen concentration in the overlying water. (**d**) Total phosphorus removal efficiency. Initial total phosphorus concentration was 1.0 mg/L. Bars sharing the same letters are not significantly different at P = 0.05. Values are mean ± SD (n = 3).

Ammonium nitrogen removal was affected significantly by water N supply, especially at the initial period of the experiment (Fig. 5a). Percent removal of ammonium nitrogen at NH_4_^+^-N concentration 3 mg/L was significantly higher than at other concentrations in 8 d. Ammonium nitrogen removal of all treatments increased with time, reached the maxima and decreased thereafter. As shown in the Fig. 5a, it seemed to take a longer time to reach the maxima at higher NH_4_^+^-N concentrations. The maximum NH_4_^+^-N removal efficiency was more than 80% obtained at day 8 for NH_4_^+^-N less than 7 mg/L, and much over 60% at NH_4_^+^-N as high as 15 mg/L, indicating plants played an important role in the removal of NH_4_^+^-N.

As for TN removal, the removal efficiency showed an increasing trend and reached the maxima of much over 50% at day 12, and then it gradually declined with time and levelled off (Fig. 5b). The difference of removal efficiency among various NH_4_^+^-N concentration was relative small. At day 20, there was no significant difference among all N level treatments. The maxima of TN removal was between 54% to 66%, ranked as C4 > C3 > C1 > C2 > C5. Total nitrogen concentration was 2.29 mg/L at the end of the experiment at initial NH_4_^+^-N concentration of 15 mg/L, while others were below 0.5 mg/L.

Nitrate nitrogen concentration was much lower during the experiment (Fig. 5c). For initial NH_4_^+^-N concentration 3 to 10 mg/L, nitrate nitrogen concentration was less than 0.2 mg/L. However, it was much higher at NH_4_^+^-N concentration of 15 mg/L, the maxima reached 0.35 mg/L at day 16.

### Phosphorus removal in the overlying water

Total phosphorus removal varied slightly among different NH_4_^+^-N treatments, and the difference declined gradually with time (Fig. 5d). At the end of the experiment, there was no significant difference among all treatments. At the first stage of the experiment, TP removal efficiency increased with time, and reached the maximum of 42% to 57% at day 6. Then it decreased slightly and remained constant at day 20. At the end of the experiment, TP concentration of all treatments was lower than 0.1 mg/L, satisfying the Class III standard for surface water environmental quality^25^.

### Phosphorus and nitrogen contents in the sediments

**Figure 6:**
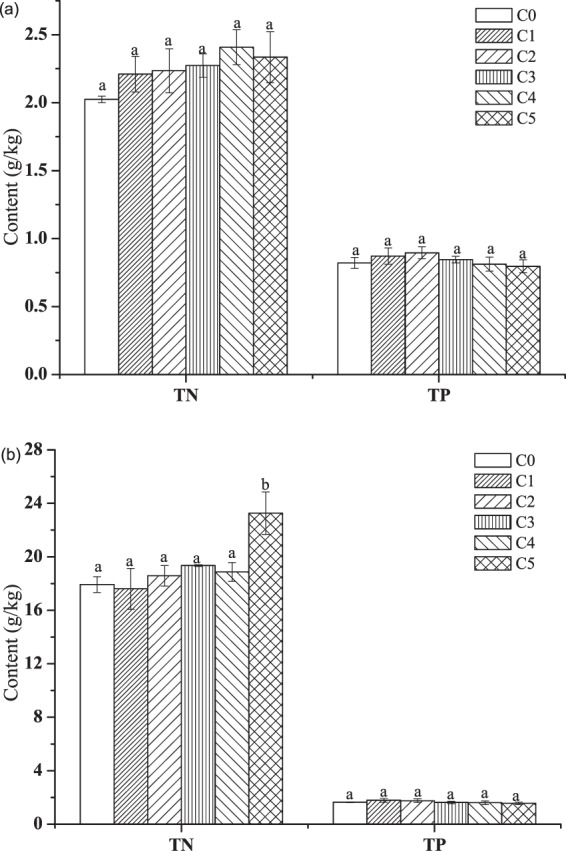
Nitrogen and P contents in the sediments (**a**) and plant tissues (**b**) before and after the trial at different NH_4_Cl concentrations (C0: initial value; C1: 3 mg/L; C2: 5 mg/L; C3: 7 mg/L; C4: 10 mg/L; C5: 15 mg/L). Initial total phosphorus concentration was 1.0 mg/L. Bars sharing the same letters are not significantly different at P = 0.05. Values are mean ± SD (n = 3).

Nitrogen content in the sediment increased with the increase of water N supply, and reached the maxima at NH_4_^+^-N concentration 10 mg/L (Fig. 6a). However, the difference between sediment at 3–15 mg/L NH_4_^+^-N was not significant (p > 0.05). Nitrogen content ranged from 2.2 to 2.5 g/kg, with an increase of 9.1% to 25%. Phosphorus in the sediments did not vary significantly among the treatments, and it was slightly higher at lower NH_4_^+^-N concentration treatments (NH_4_^+^-N concentration ≤5 mg/L).

### Nitrogen and phosphorus contents in the plants tissues

Nitrogen in the tissues of plants increased slightly with increasing water N supply at lower NH_4_^+^-N treatments (NH_4_^+^-N concentration ≤10 mg/L) as shown in Fig. 6b. It was noted that nitrogen content was significantly greater at NH_4_^+^-N 15 mg/L. The value increased by 23% when the NH_4_^+^-N concentration increased from 10 to 15 mg/L. Phosphorus in the tissues was not distinct among all treatments, and did not show significant changes from the initial value.

## Discussion

### Growth and morphology on different sediments

Sediment is an important source of nitrogen and phosphorus for submerged plants^26,27^. It has been reported that in most situations root uptake is the primary pathway for nitrogen and phosphorus^28^. However, Madsen and Gedergreen^15^ found that enrichment of sediment had no effect on the relative growth rate of *Elodea Canadensis* and *Callitriche cophocarpa* under sufficient nutrient environment. In the present study, the plant height, tiller number, and biomass accumulation were significantly greater on silt than those on gravel and sand. This indicated that *Rotala rotundifolia* preferred to grow in sediments with high nutrients. Nitrogen and P contents in the silt were 2.0 g/kg and 0.82 g/kg, respectively. The differences in the height and tiller number of plants among silt, gravel and sand sediments at initial period of the experiment were notably smaller than those in the latter period. It may be attributed to the nutrient limitation on gravel and sand sediments in the latter period. The experiment was conducted in relative static mode, and no additional nutrient was provided. Therefore, the plants grown on sand and gravel sediments may not obtain sufficient nutrients as the nutrients in the overlying water gradually reduced with time. However, the plants in the silt treatment could obtain continuous nutrients from the rich sediments during the latter stage. As for similar mineral sediments, the average height increment of plants on sand sediment was 8.4% higher than that on gravel sediment at the end of experiment. The results were consistent with the observations in other freshwater submerged plants^29^. The reason for this is that the grain size of sediment can be important in determining the rooting depth of species given the different abilities of plants to physically penetrate sediments^30^.

### Nutrient removal on silts with different water N supply

*Rotala rotundifolia* grew well as the NH_4_^+^-N concentration varied from 3 to 15 mg/L. Phenomenon of yellow leaves, stems, rotted roots did not appear in all treatments during the experiment. Total biomass increased with increasing NH_4_^+^-N concentration in the range of 3 to 10 mg/L. However, further increase of NH_4_^+^-N concentration up to 15 mg/L, total biomass decreased and the value even lower than that at NH_4_^+^-N concentration of 3 mg/L. Similar results have previously been observed in other submerged aquatic plants^31,32^. Higher nutrient availabilities enhanced plant growth and biomass accumulation, but excessive NH_4_^+^-N can result in toxicity in plants when cultured on NH_4_^+^-N as exclusive N-source^33^. Wang *et al*.^34^ and Su *et al*.^35^ observed oxidative stress and inhibition of photosynthesis on plant leaves at NH_4_^+^-N concentration more than 10 mg/L.

The effect of NH_4_^+^-N concentration in water differs widely among plant species. Sensitive species show chlorosis of leaves and suppression of growth at external NH_4_^+^-N concentrations above 0.1–0.5 mM^36^. However, *Salvinia natans* grew well at external NH_4_^+^-N concentrations up to 5 mM^33^. *Rotala rotundifolia* can be tolerant to NH_4_^+^-N concentration as high as 10 mg/L. Even at 15 mg/L NH_4_^+^-N, it still grew well, although the growth rate slowed down.

Nitrogen removal by plants at the initial period of the experiment was lower at higher NH_4_^+^-N, but increased with time, as shown in Fig. 5. At the end of the experiment, differences of nitrogen removal among various NH_4_^+^-N concentrations were much smaller. There was no significant difference in TN removal among all treatments at 20 d. This result may be attributed to the adaptation of plant to higher NH_4_^+^-N. It also revealed the synergistic effect of plant on nitrification/denitrification note that the removal efficiencies in the figures were calculated by the values of treatment groups minus those of control. The nitrate nitrogen concentration was significantly higher at NH_4_^+^-N concentration 15 mg/L than other treatments.

Several studies have indicated that volatilization, plant uptake and nitrification/denitrification are the major mechanisms for nitrogen removal^37,38^. Since the pH of the water of all treatments was between 6.35–8.0 during the experiment, the shift from NH_4_^+^ to NH_3_ gas was not significant. The contribution of direct uptake by plants and synergistic effect of plants were calculated. Uptake by plants increased at low N levels (NH_4_^+^-N concentration ≤7 mg/L), but decreased when NH_4_^+^-N concentration increased further. Particularly, uptake by plants decreased sharply from 32% to 16% when NH_4_^+^-N concentration increased from 10 mg/L to 15 mg/L. It might have mainly been attributed to the sharp reduction in biomass as shown in Table 1. The contribution of uptake by plants ranged from 16% to 39%, which was similar with *Potamogeton crispus* reported by Li *et al*.^39^. However, direct assimilation by plants varies with considerations of other factors such as density and climate^40,41^.

Synergistic effect of plants was significant at higher water N levels, and they were 28% and 39% at NH_4_^+^-N concentrations of 10 and 15 mg/L respectively. The synergistic effect of plants was much higher than assimilation by plants at NH_4_^+^-N concentration 15 mg/L, which was in line with the findings obtained by Jin *et al*.^42^. There is a redox micro-interface around the stems and leaves of submerged macrophyte, which provides organic materials for nitrifiers^43^. Korner^44^ observed considerable numbers of autotrophic and heterotrophic nitrifying and denitrifying bacteria in the epiphytic communities of different species of submerged macrophytes of a treated sewage channel. It was concluded that epiphytic nitrifiers were important for the total nitrification as nitrifiers in the sediment.

Phosphorus removal in the overlying water was enhanced with increasing water N supply at the initial period of the experiment. However, it did not show significant difference after 12 days. The P contents in the plants tissues were slightly affected by water N supply. Similar results were also reported in previous studies^45,46^. These results may suggest that abundant external N may not impact both P removal from water and the assimilation capacity of *Rotala rotundifolia* for P.

### Uncertainties of the present study

The present study was indoor experiment under control environment. However, the growth and purification capacity of the plant for eutrophic water are also influenced by many factors, such as sunlight, transparency, temperature, phosphorus concentration, constituents of sediment, etc. Some factors may exert direct mutual influence on each other. Thus, the performance of the plant in the field environment might be a little different. In the future study, the plant will be used in selected region of the river to investigate the long-term performance for nitrogen and phosphorus.

## Conclusions

*Rotala Rotundifolia* grew better on silt than on sand and gravel. Plant height, tiller number and biomass accumulation were significantly greater than grown on other two sediments. It was tolerant to NH_4_^+^-N level as high as 15 mg/L. Growth of *Rotala rotundifolia* was enhanced at N levels in the range of 3 to 10 mg/L, but inhibited by further increase of N concentration. Total nitrogen and TP concentrations in the overlying water at 3–10 mg/L NH_4_^+^-N were below 0.5 and 0.1 mg/L respectively at the end of experiment, satisfying the Class II and III standard for surface water environmental quality. Nitrogen contents in the sediments increased with elevated N level. However, N accumulation in the tissue of plants showed no apparent regularity, and the greatest value was obtained at NH_4_^+^-N concentration 15 mg/L. Neither P contents in the sediments nor tissues of plants were affected significantly by additional N supply. There was no significant difference in the P content of plants and sediments among all treatments. Direct uptake by plants on N removal accounted for 16% to 39% when P concentration was 1.0 mg/L. Synergistic effect of plants was significant at NH_4_^+^-N 15 mg/L. Thus, the study documented that *Rotala rotundifolia* is a promising candidate for N and P removals from waters.
